# The Functional Roles of RNAs Cargoes Released by Neutrophil-Derived Exosomes in Dermatomyositis

**DOI:** 10.3389/fphar.2021.727901

**Published:** 2021-09-17

**Authors:** Liya Li, Xiaoxia Zuo, Di Liu, Hui Luo, Honglin Zhu

**Affiliations:** ^1^ The Department of Rheumatology and Immunology, Xiangya Hospital of Central South University, Changsha, China; ^2^ The Department of Rheumatology and Immunology, The Third Xiangya Hospital, Central South University, Changsha, China; ^3^ Provincial Clinical Research Center for Rheumatic and Immunologic Diseases, Xiangya Hospital, Changsha, China; ^4^ National Clinical Research Center for Geriatric Disorders, Xiangya Hospital, Changsha, China

**Keywords:** dermatomyositis, neutrophil-derived exosome, lncRNA, miRNA, PI3K-Akt, MAPK, AMPK, FoxO

## Abstract

Dermatomyositis (DM) is an idiopathic inflammatory myopathy characterized by cutaneous manifestations. We first identified the profiles of noncoding RNAs (lncRNAs and miRNAs) in peripheral neutrophil exosomes (EXOs) of DM patients and explored their potential functional roles. Bioinformatics analyses were performed with R packages. Real-time quantitative PCR was used to validate the altered RNAs in DM neutrophil EXO-stimulated human dermal microvascular endothelial cells (HDMECs) and human skeletal muscle myoblasts (HSkMCs). In DM neutrophil EXOs, 124 upregulated lncRNAs (with 1,392 target genes), 255 downregulated lncRNAs (with 1867 target genes), 17 upregulated miRNAs (with 2,908 target genes), and 15 downregulated miRNAs (with 2,176 target genes) were identified. GO analysis showed that the differentially expressed (DE) lncRNAs and DE miRNAs participated in interleukin-6 and interferon-beta production, skeletal muscle cell proliferation and development, and endothelial cell development and differentiation. KEGG analysis suggested that DE lncRNAs and DE miRNAs were enriched in the PI3K–Akt, MAPK, AMPK and FoxO signalling pathways. Many novel and valuable DE lncRNAs and DE miRNAs interacted and cotargeted in the PI3K–Akt, MAPK, AMPK and FoxO signalling pathways. Our study suggests that neutrophil EXOs participate in DM pathogenesis through lncRNAs and miRNAs in the PI3K–Akt, MAPK, AMPK and FoxO signalling pathways.

## Introduction

Dermatomyositis (DM) is an idiopathic inflammatory myopathy with characteristic cutaneous manifestations and symmetrical progressive proximal weakness (Bohan and Peter, 1975a; Bohan and Peter, 1975b; Callen, 2000). The hallmark histopathological features of DM include perifascicular atrophy and inflammatory infiltration (by macrophages/neutrophils, CD20^+^ B cells, CD4^+^ T cells and plasmacytoid dendritic cells) in muscle (Dalakas, 2015; DeWane et al., 2019). Although the mechanism of DM is largely unknown, some factors are thought to contribute to DM pathogenesis, such as MHC polymorphisms, epigenetic modifications, type I interferon (IFN) signalling, myositis-specific antibodies and many other pathways, such as the phosphoinositol-3-kinase (PI3K)–v-akt murine thymoma viral oncogene homologue (Akt), mitogen-activated protein kinase (MAPK), adenosine monophosphate-activated protein kinase (AMPK) and Forkhead box O (FoxO) signalling pathways (Lahoria et al., 2016; Gao et al., 2017; Tartar et al., 2018; Gao et al., 2019).

In recent years, neutrophils have been recognized as participants in imbalances among autoimmune responses directed at tissue-specific antigens with the ability to inhibit and control these responses (Wahren-Herlenius and Dörner, 2013) and as intermediates between effectors and regulatory mechanisms (Navegantes et al., 2017). Neutrophils play pathogenic roles by releasing various molecules, such as proteases, cytokines, reactive oxygen species and exosomes (EXOs), extracellularly after triggering the immune complex (Glennon-Alty et al., 2018). Neutrophils also interact with macrophages, dendritic cells, B and T cells, and natural killer cells that produce IFN-γ, regulating the immune response (Jaillon et al., 2013). In DM patients, an increased baseline peripheral blood neutrophil-to-lymphocyte ratio is associated with pulmonary involvement, disease activity and worse overall survival (Yang et al., 2017; Gao et al., 2018a; Ha et al., 2018). In our previous study, proteinases and proteins in the neutrophil cytoplasm were found to play important roles in muscle inflammatory cell infiltration and vascular damage in DM patients (Gao et al., 2018b; Xiao et al., 2019). All of the above results suggest that neutrophils play important roles in the pathogenesis of DM; however, the detailed mechanisms need further research.

EXOs are extracellular vesicles (EVs) that are 30–150 nm in diameter and are secreted by most cells. EXOs express specific surface markers, including tumour susceptibility gene 101, integrins and tetraspanins (CD63, CD9, and CD81/82) (Yáñez-Mó et al., 2015). EXOs contain selective cargoes of RNAs, DNAs and proteins, which are valuable sources of intercellular signalling molecules, biomarkers and treatment targets (Li et al., 2018; Li et al., 2019). For example, serum EXOs can restore cellular function *in vitro* and can be employed for the treatment and noninvasive diagnosis of dysferlinopathy (Dong et al., 2018). In addition, EXOs released from inflamed myotubes induce myoblast inflammation and inhibit myogenic mechanisms while stimulating atrophic signals (Kim et al., 2018). Neutrophil-derived EXOs (neutrophil EXOs) also play important roles in immune responses (Rossaint et al., 2016). In generalized pustular psoriasis, proteomic analysis of neutrophil EXO contents has identified olfactomedin 4 as the critical differentially expressed (DE) protein that mediates autoimmune inflammatory responses (Shao et al., 2019). In the progression of asthma, neutrophil EXOs can modulate immune responses, enhance the proliferation of airway smooth muscle cells, and promote airway remodelling (Vargas et al., 2016). In chronic obstructive pulmonary disease (COPD), activated neutrophil EXOs predominate in lung fluids. These EXOs can degrade extracellular matrix by protecting surface neutrophil elastase from alpha-1 antitrypsin inhibition. Transferring activated neutrophil EXOs causes a COPD-like phenotype in murine lungs in which alveoli are destroyed via neutrophil elastase (Genschmer et al., 2019). In our previous study, neutrophil EXOs from systemic sclerosis patients were found to inhibit the proliferation and migration of human dermal microvascular endothelial cells (HDMECs) (Li et al., 2020a; Li et al., 2020b). Therefore, we hypothesized that neutrophil EXOs might lead to muscle damage in DM patients.

Recently, noncoding RNA regulatory mechanisms involving microRNAs (miRNAs, <50 nucleotides) and long noncoding RNAs (lncRNAs, >200 nucleotides) have been widely studied in the context of DM (Peng et al., 2016; Gao et al., 2019; Mazzone et al., 2019). Many miRNAs and lncRNAs are associated with disease activity and participate in the pathogenesis of DM (Satoh et al., 2005; Misunova et al., 2016; Peng et al., 2016). In juvenile DM (JDM) patients, the miRNAs of plasma EXOs are capable of altering transcriptional programmes within endothelial cells (Jiang et al., 2019). Here, we analysed the noncoding RNAs (lncRNAs and miRNAs) profiles of neutrophil EXOs from DM patients and sought insights into their predicted functional roles, such as their roles in pathogenetic mechanisms, and their potential utility as new biomarkers and therapeutic targets.

## Materials and Methods

### Patients and Controls

We collected blood from 20 DM patients and 22 normal controls (NCs) who were all Han Chinese. Among these participants, 5 patients with DM as primary diagnosis and 5 age- and sex-matched NCs were selected for lncRNA and miRNA sequencing analyses. The clinical characteristics of 5 DM patients for neutrophil EXOs RNAseq are shown in Supplementary Table S1. All patients fulfilled the 1975 Bohan and Peter diagnostic criteria for DM and were enrolled at the Department of Rheumatology and Immunology at the Xiangya Hospital of Central South University (Bohan and Peter, 1975a; Bohan and Peter, 1975b). The clinical characteristics of the participants, including their demographic characteristics, serological characteristics, organ involvement and medications, are shown in Supplementary Table S2.

### Isolation and Culture of Neutrophils

Peripheral whole blood samples (10 ml) were collected in EDTA anticoagulant-coated tubes and processed within 2 h. Neutrophils were isolated by density gradient equilibrium centrifugation using Histopaque-1077 and Histopaque-1119 (Sigma-Aldrich, St. Louis, United States) according to the manufacturer’s instructions. The neutrophil pellets were used for culture or stored at −80°C until analysis. For neutrophil culture, the cell pellets (cell viability>90%) were resuspended in the appropriate volume (2–5×10^6^ cell/ml) of 1,640 medium (Gibco, Carlsbad, United States) supplemented with 10% EXO-free foetal bovine serum (FBS, Gibco), which was prepared by collecting the supernatant after ultracentrifugation of FBS at 150,000 × *g* for 3 h at 4°C (the same procedure was used for the EXO-free FBS described below). The neutrophils were incubated for 2 h at 37°C in a humidified atmosphere containing 5% CO_2_. The cell pellets and supernatants were collected separately for further analysis.

### Isolation and Identification of Exosomes and EXO RNAs

We isolated EXOs from cultured neutrophil supernatants using an exoEasy Maxi Kit (Qiagen, Frederick, United States) according to the manufacturer’s instructions. The neutrophil EXOs were dissolved in 600 μl of eluting buffer and stored at −80°C until analysis. Neutrophil EXOs were identified as cup-shaped double-membrane structures by transmission electron microscopy (FEI Tecnai G2 Spirit Twin, Hillsboro, United States) via negative staining (Supplementary Figure S1A), were confirmed to express CD63 with a FACSCalibur flow cytometer (BD Biosciences, San Jose, United States) using EXOome-Human CD63 Isolation/Detection Dynabeads (Invitrogen, Thermo Fisher Scientific, Lithuania) and anti-human CD63-PE antibodies (12–0,639, eBioscience, United States) according to the manufacturer’s protocol (Supplementary Figure S1B) and were verified to meet the expected EXO size by dynamic light scattering with a Zetasizer Nano ZS instrument (Malvern Instrument, Marvin, United Kingdom) (Supplementary Figure S1C).

Total RNA was extracted from the neutrophil EXOs with TRIzol (Invitrogen Life Technologies, California, United States) according to the manufacturer’s protocol. An Agilent 2,200 TapeStation (Agilent Technologies, California, United States) and a Qubit® 2.0 Fluorometer (Life Technologies) were used to assess the quantity and integrity of the total RNA and the purified library products described below.

### RNA Library Construction, Sequencing and Data Analysis

RNAs libraries were created with an NEBNext® Multiplex Small RNA Library Prep Set for Illumina (NEB, Ipswich, United States) for small RNA and an NEBNext® Ultra™ RNA Library Prep Kit for Illumina for lncRNA, according to the manufacturer’s instructions. Paired-end sequencing (PE150) with the Illumina 3,000 platform was performed (RiboBio Co.,Guangzhou, China). The number of lncRNAs and miRNAs detected in neutrophil EXOs are shown in Supplementary Table S3. The sequencing reads were preprocessed with fastQC software and Trimmomatic tools (v 0.36) to assess the read quality and to filter out poor-quantity reads, HISAT2 was used to map the reads to the human reference genome hg19; uniquely mapped reads were assigned to annotated genes with HTSeq (for lncRNAs) and the Burrows-Wheeler Aligner (for miRNAs). The miRDeep2 database was used to identify known mature miRNAs based on miRBase (v21) (www.miRBase.org) and to predict novel miRNAs. DESeq2 was used to identify the significantly DE lncRNAs (adjusted *p* < 0.05 and |log2(fold change)| > 1.5), while the R package edgeR was used to identify the significantly DE miRNAs (adjusted *p* < 0.05 and |log2(fold change)| > 1).

### Bioinformatics Analysis for Differentially Expressed lncRNAs and Differentially Expressed miRNAs

LncRNAs regulate potential target genes through two major mechanisms: cis regulation and trans regulation. Target genes in cis were obtained by integrating the DE lncRNAs and their adjacent (within 10 kb) DE mRNAs. After extracting the sequences of the DE lncRNAs and DE mRNAs, both BLAST software (for initial screening) and RNAplex software (for re-screening) were used to identify potential target genes of the lncRNAs in trans. The target genes for selected miRNAs were predicted with the targetScan, miRDB, miRTarBase and miRWalk software programs. Moreover, the miRanda, PITA and RNAhybrid software programs were used to identify common elements between the lncRNAs and miRNAs and to predict the miRNAs associated with lncRNAs of interest.

The target genes of the DE lncRNAs and DE miRNAs were subjected to pathway analysis with the Gene Ontology (GO) (http://geneontology.org/) and Kyoto Encyclopedia of Genes and Genomes (KEGG) (https://www.genome.jp/kegg) databases. GO and KEGG enrichment analyses were performed with the R package clusterProfiler (Yu et al., 2012). Interaction and coexpression networks for lncRNAs, miRNAs and mRNAs were constructed with Cytoscape software (http://apps.cytoscape.org/).

### Cell Culture

HDMECs were obtained from Cell Biolabs (# CBR130858, San Diego, United States) and cultured in DMEM (Gibco) with 10% EXO-free FBS. Human skeletal muscle myoblasts (HSkMCs) were purchased from ScienCell and cultured in Skeletal Muscle Cell Medium (ScienCell, San Diego, United States) with 5% EXO-free FBS. All cells were cultured at 37°C in a 5% CO_2_ humidified incubator. Both HDMECs and HSkMCs were used when they reached 60–70% confluence and were then stimulated with 20% neutrophil EXOs (volume concentration, 160 µl of neutrophil EXO eluting buffer and 640 µl of medium) per well for 48 h in 24-well plates. Each experiment was repeated three times with three samples per group.

### Validation Using Real-Time Quantitative PCR

Total RNA from neutrophils, HDMECs and HSkMCs was extracted using TRIzol according to the manufacturer’s instructions. For lncRNAs, cDNA was prepared using a PrimeScript™ RT Reagent Kit (Perfect Real Time, Takara, Japan). Real-time quantitative PCR was performed using TB Green PCR Master Mix (Takara, Japan) on a 7,500 Real-Time PCR System (Applied Biosystems, California, United States). *GAPDH* expression was used as the endogenous control. The gene-specific primers are shown in Supplementary Table S4. The relative expression of miRNAs was measured with a miDETECT A Track™ miRNA qRT-PCR Starter Kit (RiboBio.Co., Guangzhou, China) on the 7,500 Real-Time PCR System. The miRNA-specific primers and the Uni-Reverse Primer were purchased from RiboBio Co. (Guangzhou, China). The miRNA primers product information is shown in Supplementary Table S5. U6B small nuclear RNA was used as the endogenous control to normalize the sample data. Each group had more than six samples.

### Statistical Analysis

All data except for the RNA sequencing data were analysed with GraphPad Prism 5 software, and statistical significance was set as a two-sided *p* < 0.05. Numerical variables with a normal distribution are presented as the mean ± SEM and were analysed by unpaired t-test. Data with a nonnormal distribution are shown as the median and were assessed with the Wilcoxon rank sum test.

## Results

### Differentially Expressed lncRNAs in Dermatomyositis Neutrophil Exosomes

We identified 379 DE lncRNAs in neutrophil EXOs in DM patients compared with NCs, including 124 upregulated and 255 downregulated lncRNAs [Supplementary Data S1]. The top 30 DE lncRNAs are shown in Table 1 and Supplementary Table S6. Bioinformatics analysis identified 1,392 target genes for the upregulated lncRNAs and 1867 target genes for the downregulated lncRNAs [Supplementary Data S2]. GO analysis of these target genes revealed that the regulation of interleukin-6 production, IFN-β production and skeletal muscle cell proliferation terms were enriched; KEGG analysis indicated that the FoxO signalling pathway, endocytosis and JAK-STAT signalling pathway were involved (Table 3 and Supplementary Tables S7, S8).

**TABLE 1:** DE lncRNAs in the neutrophil exosomes of DM patients (Top 30).

lncRNAs	log_2_(FC)	*p* value	lncRNAs	log_2_(FC)	*p* value
ENST00000600489.1	5.432	0.000	NR_123718.1	−2.136	0.000
NR_136569.1	5.423	0.000	NR_135530.1	−2.337	0.000
ENST00000594492.1	3.713	0.000	NR_040001.2	−2.368	0.000
NR_003013.1	3.216	0.000	ENST00000444796.1	−2.434	0.001
ENST00000592523.1	3.207	0.000	ENST00000453561.2	−2.609	0.000
ENST00000607284.1	2.839	0.000	ENST00000593824.1	−3.242	0.000
NR_024393.1	2.633	0.001	ENST00000503403.1	−3.375	0.000
ENST00000526936.1	2.163	0.001	ENST00000572850.1	−3.755	0.000
ENST00000428280.1	1.970	0.001	NR_110815.1	−4.137	0.001
ENST00000419196.1	1.737	0.001	ENST00000519840.1	−4.254	0.000
ENST00000503469.2	1.577	0.001	ENST00000587907.1	−4.275	0.000
NR_135626.1	−1.764	0.001	NR_104232.1	−4.649	0.000
ENST00000438753.1	−1.899	0.000	ENST00000581910.1	−4.667	0.001
ENST00000450365.1	−1.929	0.001	ENST00000592296.1	−5.274	0.000
NR_038194.1	−2.013	0.001	ENST00000577199.1	−5.982	0.000

DE: differentially expressed; DM: dermatomyositis; FC: fold change.

### Differentially Expressed miRNAs in Dermatomyositis Neutrophil Exosomes

We identified a total of 32 DE miRNAs between DM patients and NCs from the miRNA profiles of neutrophil EXOs, including 17 upregulated and 15 downregulated miRNAs (Table 2 and Supplementary Table S9). Bioinformatics analysis predicted 2,908 target genes for the upregulated miRNAs and 2,176 target genes for the downregulated miRNAs [Supplementary Data S3]. GO enrichment analysis suggested that both of these miRNA target genes were involved in actin filament organization, muscle tissue development, endothelial cell development and differentiation and activation of MAPK activity; KEGG analysis indicated that the PI3K–Akt, MAPK, AMPK, and FoxO signalling pathways, among others, were enriched (Table 3 and Supplementary Tables S10, S11).

**TABLE 2:** DE miRNAs in the neutrophil exosomes of DM patients (*n* = 32).

miRNAs	log_2_(FC)	*p* value	miRNAs	log_2_(FC)	*p* value
hsa-miR-3614-5p	4.262	0.000	hsa-miR-1273h-3p	3.138	0.049
hsa-miR-1180-3p	4.250	0.007	hsa-miR-4792	−2.993	0.002
hsa-miR-451a	1.705	0.014	hsa-miR-1323	−2.938	0.005
hsa-miR-23a-5p	2.905	0.014	hsa-miR-516a-5p	−3.424	0.005
hsa-miR-183-5p	1.296	0.015	hsa-miR-512-3p	−3.163	0.008
hsa-miR-486-3p	2.303	0.016	hsa-miR-372-3p	−12.485	0.010
hsa-miR-223-5p	1.286	0.020	hsa-miR-4488	−3.933	0.022
hsa-miR-1268a	12.006	0.022	hsa-let-7f-1-3p	−12.101	0.027
hsa-miR-424-3p	1.291	0.030	hsa-miR-520a-3p	−2.520	0.030
hsa-miR-16-2-3p	1.031	0.031	hsa-miR-424-5p	−1.967	0.033
hsa-miR-363-3p	1.394	0.033	hsa-miR-542-3p	−2.116	0.039
hsa-miR-122-5p	1.251	0.036	hsa-miR-4433b-5p	−12.189	0.039
hsa-miR-1278	11.949	0.039	hsa-miR-1307-5p	−3.503	0.043
hsa-miR-548ad-5p	3.300	0.044	hsa-miR-195-5p	−1.511	0.046
hsa-miR-548ae-5p	3.300	0.044	hsa-miR-27b-3p	−1.632	0.047
hsa-miR-182-5p	1.099	0.045	hsa-miR-518e-3p	−3.719	0.047

DE: differentially expressed; DM: dermatomyositis; FC: fold change.

**TABLE 3:** GO and KEGG enrichment analysis for target genes of DE lncRNAs and DE miRNAs in DM neutrophil exosomes.

GO enrichment for target genes of DE lncRNAs	*p* value (up/down^a^)
regulation of interleukin-6 production	0.013/0.015
regulation of interferon-beta production	0.011/0.002
regulation of skeletal muscle satellite cell proliferation	0.045/0.019
**KEGG enrichment for target genes of DE lncRNAs**	***p* value (up/down^a^)**
FoxO signaling pathways	0.037/0.247
Endocytosis	0.049/0.214
JAK-STAT signaling pathway	0.153/0.013
**GO enrichment for target genes of DE miRNAs**	***p* value (up/down^a^)**
actin filament organization	0.000/0.001
muscle tissue development	0.000/0.000
endothelial cell development and differentiation	0.000/0.000
activation of MAPK activity	0.000/0.001
**KEGG enrichment for target genes of DE miRNAs**	***p* value (up/down^a^)**
PI3K-Akt signaling pathways	0.000/0.000
MAPK signaling pathways	0.000/0.000
AMPK signaling pathways	0.000/0.000
FoxO signaling pathways	0.000/0.000
Endocytosis	0.002/0.017
Regulation of actin cytoskeleton	0.001/0.000
Wnt signaling pathway	0.002/0.002
mTOR signaling pathway	0.009/0.002
TNF signaling pathway	0.012/0.001
Th17 cell differentiation	0.013/0.009
VEGF signaling pathway	0.048/0.032

a The *p* value of predicted target genes for upregulated/downregulated lncRNAs/miRNAs.

GO: Gene Ontology; KEGG: Kyoto Encyclopedia of Genes and Genomes.

DE: differentially expressed; DM: dermatomyositis.

### Interaction Relationships of Differentially Expressed lncRNAs and Differentially Expressed miRNAs in the Phosphoinositol-3-Kinase–Akt, Mitogen-Activated Protein Kinase, Adenosine Monophosphate-Activated Protein Kinase and FoxO Signalling Pathways

LncRNAs can regulate miRNAs in some cellular processes. Here, we analysed the relationships between the DE lncRNAs and DE miRNAs in DM neutrophil EXOs and found that 91 lncRNAs (58 downregulated and 33 upregulated lncRNAs) and 23 miRNAs (11 downregulated and 12 upregulated miRNAs) might interact with each other [Supplementary Figure S2]. From the GO and KEGG analyses above, we found that the PI3K–Akt, MAPK, AMPK and FoxO signalling pathways were enriched for the predicted target genes of both DE lncRNAs and DE miRNAs. Thus, we analysed the lncRNAs, miRNAs and target genes involved in these four pathways. We found that 43 predicted target genes were able to be regulated by both lncRNAs and miRNAs. Finally, we obtained an interaction network that included 50 lncRNAs, 18 miRNAs and their 43 cotarget mRNAs (Table 4). Among the relationships, 3 of the lncRNA-miRNA pairs (in which the miRNAs were regulated by lncRNAs) were NR_046101.1–hsa-miR-3614-5p, ENST00000444868.2–hsa-miR-195-5p and ENST00000433036–hsa-miR-512-3p.

**TABLE 4:** Co-target genes for DE lncRNAs and DE miRNAs in the PI3K-Akt, MAPK, AMPK, FoxO signaling pathways.

Co-target genes (*n* = 43)	DE lncRNAs (*n* = 50)	DE miRNAs (*n* = 18)
*ANGPT2*	ENST00000581633.1, NR_125914.1, NR_125915.1	hsa-miR-542-3p
*ATG12*	ENST00000584643.1	hsa-miR-183-5p
*BCL2*	ENST00000588307.1	hsa-miR-486-3p, hsa-miR-182-5p
*CACNG3*	ENST00000567624.1	hsa-miR-486-3p
*CACNG8*	**NR_046101.1****^a^**, ENST00000565495.1, ENST00000539313.1, ENST00000600508.1, ENST00000584643.1	**hsa-miR-3614-5p** **^a^**
*CPT1A*	ENST00000582536.1, NR_038219.1, ENST00000591918.2, ENST00000504658.1, ENST00000592296.1, ENST00000508878.1, ENST00000526936.1, ENST00000511517.1	hsa-miR-512-3p, hsa-miR-372-3p, hsa-miR-520a-3p
*CREB1*	ENST00000448256.1, ENST00000609726.1	hsa-let-7f-1-3p, hsa-miR-512-3p, hsa-miR-3614-5p, hsa-miR-182-5p, hsa-miR-122-5p, hsa-miR-27b-3p
*CREB3L1*	ENST00000527239.1	hsa-miR-182-5p
*CRK*	NR_104232.1, NR_039978.1, ENST00000519451.1, ENST00000609726.1, ENST00000544086.1, ENST00000539313.1	hsa-miR-372-3p, hsa-miR-195-5p, hsa-miR-424-5p
*EEF2K*	ENST00000510274.1, ENST00000504658.1, ENST00000428280.1, ENST00000584643.1, ENST00000591918.2	hsa-miR-1323
*ELK4*	ENST00000582269.1	hsa-miR-3614-5p, hsa-miR-520a-3p, hsa-miR-195-5p, hsa-miR-424-5p, hsa-miR-372-3p
*ERBB3*	NR_125423.1, ENST00000581633.1	hsa-miR-512-3p
*FGF5*	ENST00000582269.1	hsa-miR-542-3p
*FGFR1*	NR_125423.1, ENST00000544086.1	hsa-miR-424-5p, hsa-miR-486-3p
*FGFR2*	ENST00000444868.2, ENST00000585761.1	hsa-miR-1323
*FLT1*	ENST00000609726.1, ENST00000539313.1, ENST00000581633.1	hsa-miR-372-3p
*FOXO3*	ENST00000444868.2	hsa-miR-182-5p
*GHR*	ENST00000606096.1	hsa-miR-195-5p
*GRB2*	NR_134920.1, ENST00000585921.1, ENST00000609726.1	hsa-miR-1323
*IL1R1*	ENST00000544086.1	hsa-miR-1323
*IL2RA*	NR_038219.1	hsa-miR-122-5p
*IL6R*	ENST00000592296.1, ENST00000584643.1	hsa-miR-3614-5p, hsa-miR-451a
*IRAK4*	NR_039978.1, NR_125915.1, NR_125914.1	hsa-miR-372-3p, hsa-miR-520a-3p, hsa-miR-512-3p
*ITGA2*	ENST00000565495.1	hsa-miR-424-5p, hsa-miR-372-3p, hsa-miR-195-5p
*ITGB8*	NR_110119.1, ENST00000584643.1	hsa-miR-183-5p, hsa-miR-182-5p, hsa-miR-372-3p
*MAP3K13*	NR_110761.1, ENST00000399866.3, ENST00000584643.1, ENST00000436249.3	hsa-miR-183-5p, hsa-miR-542-3p
*MDM2*	ENST00000366360.2, ENST00000533008.1	hsa-miR-542-3p, hsa-miR-3614-5p, hsa-miR-1278
*MEF2C*	NR_109940.1, ENST00000510274.1	hsa-miR-182-5p, hsa-miR-183-5p
*MRAS*	NR_023922.2	hsa-miR-424-5p
*NFATC3*	ENST00000569088.1	hsa-miR-512-3p
*NR4A1*	NR_037177.1, ENST00000462717.1, ENST00000568362.1, ENST00000433036.1	hsa-miR-542-3p
*OSMR*	NR_110,761.1, ENST00000425192.1, ENST00000428280.1, ENST00000558478.1, ENST00000584643.1	hsa-miR-1323
*PIK3CD*	ENST00000591918.2	hsa-miR-4792
*PIK3R2*	ENST00000585921.1	hsa-miR-486-3p
*PPM1B*	ENST00000585761.1	hsa-miR-1323, hsa-miR-182-5p
*RAP1A*	NR_023922.2	hsa-miR-512-3p
*RPS6KA3*	NR_125423.1, NR_134920.1, NR_039978.1, NR_125914.1, NR_125915.1, ENST00000591854.1, ENST00000581633.1, ENST00000582536.1, **ENST00000444868.2** **^b^**	hsa-miR-183-5p, hsa-miR-372-3p, **hsa-miR-195-5p** **^b^**
*SLC2A4*	**ENST00000433036.1** **^c^**	hsa-miR-520a-3p,**hsa-miR-512-3p** **^c^**
*SMAD2*	ENST00000582269.1, ENST00000606096.1, ENST00000462717.1, ENST00000366360.2	hsa-miR-1278
*SMAD4*	NR_023922.2, ENST00000510274.1, ENST00000568362.1	hsa-miR-27b-3p, hsa-miR-183-5p, hsa-miR-182-5p
*STAT3*	ENST00000526936.1, ENST00000444868.2	hsa-miR-486-3p
*STMN1*	ENST00000582269.1, ENST00000366360.2, ENST00000462717.1	hsa-miR-1268a
*THEM4*	NR_046101.1, NR_038219.1	hsa-miR-512-3p, hsa-miR-183-5p, hsa-miR-372-3p

a, b, c The lncRNA-miRNA pairs of interaction relationship.

DE: differentially expressed.

### Analysis of Differentially Expressed lncRNAs and Differentially Expressed miRNAs in the Phosphoinositol-3-Kinase–Akt, Mitogen-Activated Protein Kinase, Adenosine Monophosphate-Activated Protein Kinase and FoxO Signalling Pathways in Neutrophils and Neutrophil EXO–Stimulated Human Dermal Microvascular Endothelial Cells and Human Skeletal Muscle Myoblasts

**FIGURE 1:**
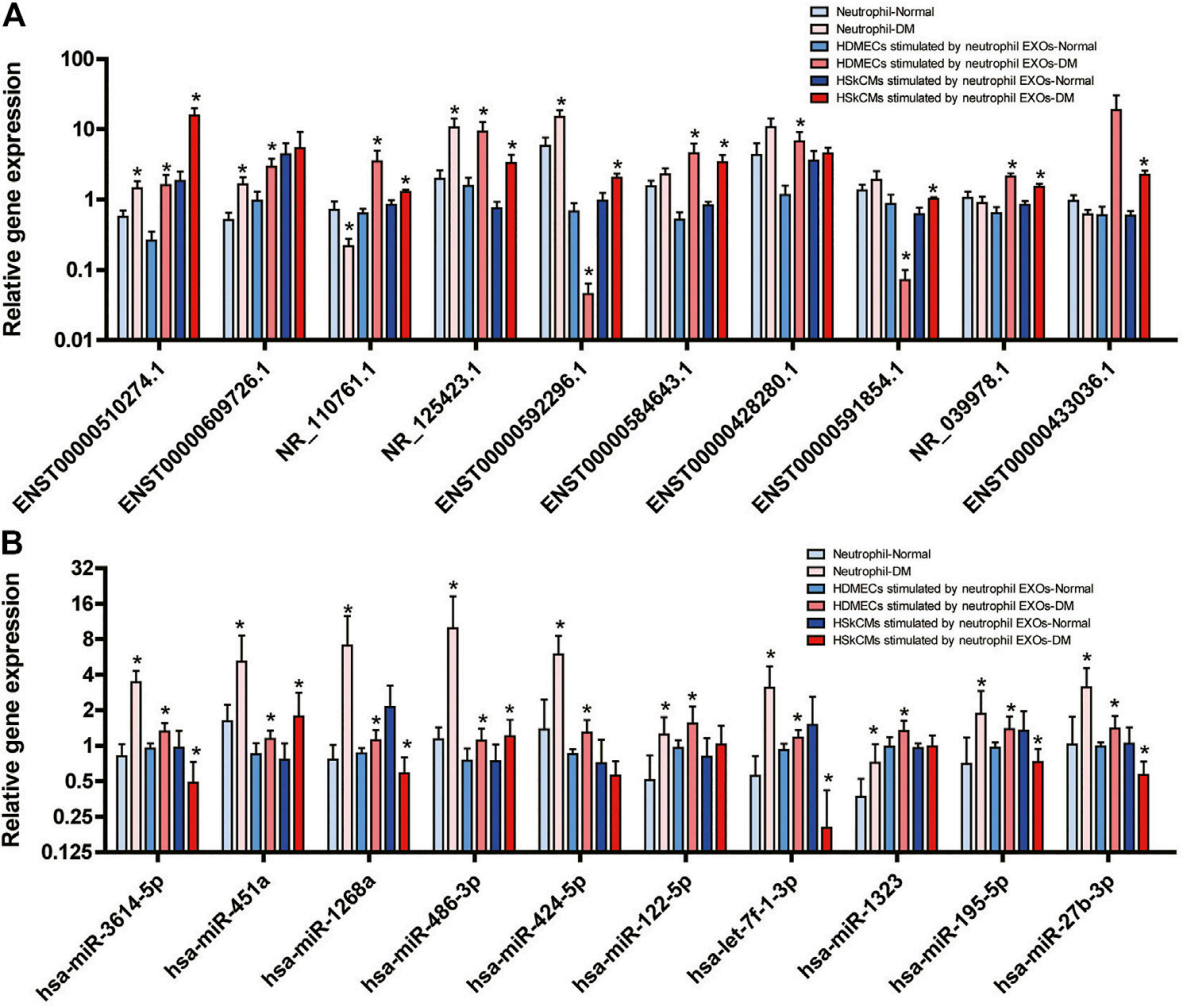
Expression levels of DE lncRNAs **(A)** and DE miRNAs **(B)** in the PI3K–Akt, MAPK, AMPK and FoxO signalling pathways validated by real-time quantitative PCR in neutrophils of DM patients and in HDMECs and HSkMCs after stimulation with DM neutrophil EXOs (*DM vs. NC).

Skeletal muscle cells and endothelial cells are target cells in the pathogenesis of DM (Yáñez-Mó et al., 2015; Gao et al., 2018b; Gao et al., 2019). To further analyze the functions of neutrophil EXOs, we validated the expression of DE lncRNAs and DE miRNAs in neutrophils and in neutrophil EXO-stimulated HDMECs and HSkMCs. As shown in Figure 1 and Supplementary Figure S3, 10 DE lncRNAs (6 upregulated and 4 downregulated lncRNAs) and 10 DE miRNAs (5 upregulated and 5 downregulated miRNAs) involved in the PI3K–Akt, MAPK, AMPK and FoxO signalling pathways were chosen for validation analysis.

Nine of the 10 DE lncRNAs (ENST00000510274.1, ENST00000609726.1, NR_110761.1, NR_125423.1, ENST00000592296.1, ENST00000584643.1, ENST00000428280.1, ENST00000591854.1 and NR_039978.1) were significantly DE in HDMECs-stimulated by neutrophil EXOs, ENST00000433036.1 was not. Eight of the lncRNAs were significantly DE in HSkMCs-stimulated by neutrophil EXOs, and five of them were DE in neutrophils. The results for most of them were consistent with the results of the lncRNA profile analysis (Figure 1A).

Among the 10 DE miRNAs, all of them (hsa-miR-3614-5p, hsa-miR-451a, hsa-miR-1268a, hsa-miR-486-3p, hsa-miR-424-5p, hsa-miR-122-5p, hsa-let-7f-1-3p, hsa-miR-1323, hsa-miR-195-5p, and hsa-miR-27b-3p) were expressed at substantially higher levels in the DM group than in the NC group for both neutrophils and HDMECs-stimulated by neutrophil EXOs. Among them, hsa-miR-3614-5p, hsa-miR-451a, hsa-miR-1268a, hsa-miR-486-3p, and hsa-miR-122-5p were significantly DE consistent with the results of the miRNA profile analysis. Among HSkMCs-stimulated by neutrophil EXOs, the expression levels of hsa-miR-451a and hsa-miR-486-3p were higher in the DM group than in the NC group, while those of hsa-miR-3614-5p, hsa-miR-1268a, hsa-let-7f-1-3p, hsa-miR-195-5p and hsa-miR-27b-3p, were lower (Figure 1B).

## Discussion

Increasing amounts of data have recently highlighted the potential contributions of EXOs to vascular injury and muscle damage in DM pathogenesis. In the current study, 10 DE miRNAs in plasma EXOs were identified JDM patients compared with NCs. JDM-derived plasma EXOs can be taken up by human aortic endothelial cells, and induce alteration in multiple genes (59 genes) in these cells, which suggests potential mechanisms by which plasmas EXOs can ultimately target vascular tissue in JDM (Jiang et al., 2019). In addition, DM plasma-derived EVs have been found to trigger proinflammatory cytokine (IFNβ, TNFα and IL-6) release and STING signalling pathway activation in circulating immune cells. The activated STING pathway is preferentially mediated by EV-dsDNA (Li et al., 2021). DE proteins have also been found in DM serum EVs. The number of serum Plexin D1+ EVs is positively associated with muscle pain or weakness in DM patients, and correlates with the levels of aldolase, white blood cells, neutrophils and platelets. In DM patients after treatment (in clinical remission), the serum levels of Plexin D1+ EVs are significantly decreased (Uto et al., 2021). As EXOs contain multiple RNAs, DNAs, lipids, and proteins, EXOs can be selectively taken up by any neighbouring or distant cells, and reprogram the recipient cells. Thus, they have extremely strong potential to be diagnostic biomarkers and therapeutic nanocarriers, and might also participate in multiple processes of DM pathogenesis.

In this study, the noncoding RNAs profiles were also changed in DM neutrophil EXOs. Functional analysis suggested that the DE lncRNAs and DE miRNAs might participate in interleukin-6 and IFN-β production, skeletal muscle cell proliferation and development, and endothelial cell development and differentiation, which are associated with DM histopathology. Our validation results and interaction analyses revealed that, many novel and valuable DE lncRNAs and DE miRNAs were cotargeted and altered in the PI3K–Akt, MAPK, AMPK and FoxO signalling pathways in the neutrophil EXO–stimulated HDMECs and HSkMCs. In the pathogenesis of DM, immune activation resulted in capillary destruction and leaded to ischemia and microinfarction, hypoperfusion, and perifascicular atrophy. Vascular endothelial cells injury was the initiating factor of DM pathogenesis, and then caused skeletal muscle damage. The functions and transcriptome levels of endothelial cells and skeletal muscle cells are different. So the reactions to neutrophil EXOs stimulation were not the same, leading to the expression of DE lncRNAs and miRNAs were not accordant. Several studies have demonstrated that many miRNAs of the PI3K–Akt–FoxO pathway participate in the posttranscriptional regulation of skeletal muscle genes, including miR-19b-3p, miR-99a-5p, miR-100-5p, miR-222-3p, miR-324-3p, and miR-486-5p (Urbánek and Klotz, 2017; Brown and Webb, 2018).

PI3K–Akt, MAPK and AMPK are major effectors of insulin metabolic action and are essential for glucose homeostasis (Schultze et al., 2012). Once the insulin/insulin-like growth factor (IGF) signalling pathway is stimulated, insulin and IGF receptors are autophosphorylated and recruit downstream PI3K–Akt, MAPK and AMPK signalling pathway members. PI3K is a lipid kinase that generates phosphatidylinositol triphosphate and subsequently activates Akt, a serine/threonine kinase that converts extracellular stimuli into a wide range of cellular responses. The MAPK family is also a protein serine/threonine kinase family that includes c-Jun N-terminal kinase (JNK), p38 MAPK and extracellular-regulated protein kinases. AMPK is mainly known as a conserved and ubiquitously expressed energy sensor (that senses increases in AMP:ATP and ADP:ATP ratios) in eukaryotic cells. In addition to their diverse functions in cellular metabolism, the PI3K–Akt, MAPK and AMPK pathways have also been widely involved throughout evolution in regulating physiological processes such as gene expression, mitosis, motility, proliferation, survival, apoptosis and differentiation, and they might be attractive therapeutic targets for diabetes, cancers and autoimmune diseases (Cargnello and Roux, 2011; Mayer and Arteaga, 2016; Olivier et al., 2018).

FoxO transcription factors are conserved regulators of a variety of cellular processes, including cell cycle regulation, redox balance, proteostasis, apoptosis, metabolism and DNA damage repair. In all species, FoxO transcription factors are subject to extensive posttranslational modification, including phosphorylation, acetylation, ubiquitination and methylation (Link, 2019). Such modification of FoxO activity can be regulated by the PI3K–Akt, MAPK and AMPK signalling pathways upon stimulation by growth factor signalling, oxidative and genotoxic stress, and nutrient deprivation (Brown and Webb, 2018). The complex PI3K–Akt, MAPK, AMPK and FoxO signalling networks play decisive roles in the maintenance of skeletal muscle homeostasis and regulate the differentiation, proliferation and regeneration of muscle cells (Wu et al., 2017; Tia et al., 2018), either together or separately.

According to studies on DM pathogenesis, skeletal muscle inflammation induces muscle atrophy by inhibiting PI3K–Akt–mediated myogenic signals, which are activated by AMPK, p38 MAPK, JNK, mTOR, and IGF-1 (Stitt et al., 2004; Li et al., 2005; Kim et al., 2017; Kim et al., 2018). In a mouse model of autoimmune myositis and in differentiated C2C12 myotubes, cellular immune stimulation and intracellular β-amyloid (Aβi) have been found to potentially independently drive muscle atrophy through the PI3K–Akt–FoxO pathways (Lee et al., 2012). Furthermore, EXOs released from inflammatory C2C12 myotubes likely contribute to inflammation-induced muscle atrophy (Kim et al., 2018). These findings suggest that the PI3K–Akt, MAPK, AMPK and FoxO signalling pathways play remarkable roles in muscle inflammation and damage in DM, possibly even through EXOs.

At present, inhibitors of PI3K–Akt (mTOR and ETP-45658), AMPK (doxorubicin hydrochloride and dorsomorphin dihydrochloride) and MAPK (PD98059, PD184352 and PD0325901) are already being successfully used in the clinic for the treatment of diverse cancer types and inflammatory diseases; in contrast, the AMPK activator metformin is the only drug targeting protein kinase activity that is widely used today, demonstrating the dual roles of AMPK activation (Arana-Argáez et al., 2010; Farhan et al., 2017). The findings of the current study might provide important insights to aid in the search for new DM therapeutics.

## Conclusion

This study identified the noncoding RNAs profiles of neutrophil EXOs. Bioinformatics analysis showed that the predicted target genes of DE lncRNAs and DE miRNAs were enriched in the PI3K–Akt, MAPK, AMPK and FoxO signalling pathways, which suggests their roles in the pathogenesis of DM. These molecules may be useful diagnostic and prognostic biomarkers in the future.

## Data Availability

The bioinformatics analysis datasets presented in this study can be found in the article/Supplementary Material. The raw data for RNAs sequencing are provided in the repository, the accession number is GSE155281.

## Abbreviations

DM, dermatomyositis; IFN, interferon; PI3K, phosphoinositol-3-kinase; Akt, v-akt murine thymoma viral oncogene homologue; MAPK, mitogen-activated protein kinase; AMPK, adenosine monophosphate-activated protein kinase; FoxO, Forkhead box O; EXOs, exosomes; EVs, extracellular vesicles; HDMECs, human dermal microvascular endothelial cells; miRNAs, microRNAs; lncRNAs, long noncoding RNAs; JDM, juvenile DM; NCs, normal controls; DE, differentially expressed; GO, Gene Ontology; KEGG, Kyoto Encyclopedia of Genes and Genomes; HSkMCs, human skeletal muscle myoblasts; IGF, insulin-like growth factor.
