# Supplementary material for: The Functional Roles of RNAs Cargoes Released by Neutrophil-Derived Exosomes in Dermatomyositis
Source: Front Pharmacol. 2021 Sep 17;12:727901. doi: 10.3389/fphar.2021.727901 (PMC8484304; doi:10.3389/fphar.2021.727901)
Supplement: Supplementary file 1 [file DataSheet4.docx]

Supplementary Table S1: Clinical characteristics of 5 DM patients for neutrophil EXOs RNAseq

| Clinical characteristics /Sample | DM1 | DM2 | DM3 | DM4 | DM5 |
| --- | --- | --- | --- | --- | --- |
| Sex/Age | M/43 | M/29 | F/63 | M/47 | F/55 |
| Diseases duration (months) | 1 | 2 | 3 | 10 | 6 |
| Serological characteristics |  |  |  |  |  |
| Anti-Jo-1 (positive) | 0 | 1 | 0 | 0 | 0 |
| Anti-Ro52 (positive) | 0 | 1 | 0 | 0 | 0 |
| Other MSAs | Anti-NXP2 | 0 | Anti-TiFiγ | 0 | Anti-TiFiγ |
| ALT (U/L) | 36.2 | 252.7 | 43.8 | 38.1 | 19.8 |
| AST (U/L) | 48.1 | 349.8 | 37.6 | 67.6 | 52.1 |
| LDH (U/L) | 569 | 903 | 358 | 524 | 553 |
| CK (U/L) | 1855.9 | 5897.1 | 169.6 | 640 | 168.6 |
| ESR (mm/h) | 50 | 80 | 27 | 116 | 55 |
| CRP (mg/L) | 32.7 | 10.1 | 3.12 | 16.4 | 11.1 |
| C3 (mg/L) | 695 | 792 | 718 | 833 | 891 |
| C4 (mg/L) | 224 | 182 | 215 | 180 | 181 |
| IgG (g/L) | 9.93 | 18.74 | 11.3 | 24 | 7.74 |
| IgM (mg/L) | 2410 | 1530 | 1190 | 1270 | 1990 |
| IgA (mg/L) | 2590 | 6530 | 1530 | 2920 | 1460 |
| Organ involvements |  |  |  |  |  |
| Intersttitial lung disease | 0 | 1 | 0 | 1 | 0 |

Supplementary Table S2: Clinical characteristics of DM patients and normal controls

| Clinical characteristic | Normal controls (n=22) | DM patients (n=20) |
| --- | --- | --- |
| Age (Mean±SEM) | 43.41±2.836 | 49.70±3.343 |
| Sex (M/F) | 9/13 | 6/14 |
| Diseases duration (months) | / | 4.350±0.6739 |
| Serological characteristics |  |  |
| Anti-Jo-1 (positive) | / | 1 |
| Anti-Ro52 (positive) | / | 9 |
| LDH (U/L) | / | 433.4±35.23 |
| CK (U/L) | / | 1256±381.2 |
| ESR (mm/h) | / | 59.58±7.411 |
| C3 (mg/L) | / | 804.6±26.91 |
| C4 (mg/L) | / | 211.4±12.4 |
| IgG (g/L) | / | 14.23±1.433 |
| IgM (mg/L) | / | 1721±167.8 |
| IgA (mg/L) | / | 2508±346.5 |
| Organ involvements |  |  |
| Interstitial lung disease | 0 | 10 |
| Medications |  |  |
| Prednisone | 0 | 20 |
| Cyclophosphamide | 0 | 8 |
| Intravenous immunoglobulin | 0 | 3 |
| Methotrexate | 0 | 4 |
| Mycophenolate mofetil | 0 | 2 |
| Thalidomide | 0 | 11 |
| Azathioprine | 0 | 1 |
| Hydroxychloroquine | 0 | 6 |

Supplementary Table S3: Number of lncRNAs and miRNAs detected in neutrophil EXOs

| Sample | Number of lncRNAs detected in the sample | Number of miRNAs detected in the sample |
| --- | --- | --- |
| DM1 | 15985 | 326 |
| DM2 | 21561 | 382 |
| DM3 | 18990 | 456 |
| DM4 | 19317 | 403 |
| DM5 | 18940 | 393 |
| NC1 | 20592 | 360 |
| NC2 | 20906 | 391 |
| NC3 | 21472 | 262 |
| NC4 | 20917 | 397 |
| NC5 | 20309 | 418 |

Supplementary Table S4: Sequences of genes specific primers used in this study

| Gene | Forward (3’-5’) | Reverse (5’-3’) |
| --- | --- | --- |
| GAPDH | GTCTCCTCTGACTTCAACAGCG | ACCACCCTGTTGCTGTAGCCAA |
| ENST00000510274.1 | AGGCAGGAAGAAAACTGGGCT | CCGGACGAAAGGGAAAGTCC |
| ENST00000609726.1 | CTAACAAAGGCCTGAAGTGGAA | CCAATGGGCAGAATCTTCACTC |
| NR_110761.1 | TGCTGTCTCCTCCTAATACGCT | TCTTGGCAAAACCTGAATGG |
| NR_125423.1 | ACACCTGTGTAAACATTGGGAA | ACATTCAACAAAGAACAAACTCAGG |
| ENST00000592296.1 | GCTATTGGGAGAGTGGCTGAG | TCAGGCAGAGGCTGGTTTTAG |
| ENST00000584643.1 | ACTATAAGAAAGGATGAGCATGGTG | GCCTCAACCTGCCAGACTCA |
| ENST00000428280.1 | TGAGCAGTGTGAAAATGAACCA | AGCATCAGGAATCTCCCAATC |
| ENST00000591854.1 | CTGGGACTACGCAACTCGCT | GCATGTGATTCCTACAGATTGCT |
| NR_039978.1 | CTCAGGAACCCCAGACGCA | CCAAGGCTCCCACTTCTGTT |
| ENST00000433036.1 | CACTGTCTGTCCAGGAGCAATG | GCCATCTTTTAGTGATTCTCTGTGT |

Supplementary Table S5: Product information of miRNA primers used in this study

| Product ID | Product Name | Size |
| --- | --- | --- |
| miRA0000261 | miDETECT A TrackTM hsa-miR-183-5p Forward Primer | 1nmol |
| miRA0000419 | miDETECT A TrackTM hsa-miR-27b-3p Forward Primer | 1nmol |
| miRA0000421 | miDETECT A TrackTM hsa-miR-122-5p Forward Primer | 1nmol |
| miRA0000461 | miDETECT A TrackTM hsa-miR-195-5p Forward Primer | 1nmol |
| miRA0001341 | miDETECT A TrackTM hsa-miR-424-5p Forward Primer | 1nmol |
| miRA0001631 | miDETECT A TrackTM hsa-miR-451a Forward Primer | 1nmol |
| miRA1000075 | miDETECT A TrackTM hsa-miR-16-2-3p Forward Primer | 1nmol |
| miRA1000076 | miDETECT A TrackTM hsa-miR-182-5p Forward Primer | 1nmol |
| miRA1000116 | miDETECT A TrackTM hsa-let-7f-1-3p Forward Primer | 1nmol |
| miRA1000127 | miDETECT A TrackTM hsa-miR-1268a Forward Primer | 1nmol |
| miRA1000202 | miDETECT A TrackTM hsa-miR-486-3p Forward Primer | 1nmol |
| miRA1000260 | miDETECT A TrackTM hsa-miR-542-3p Forward Primer | 1nmol |
| miRA1000711 | miDETECT A TrackTM hsa-miR-512-3p Forward Primer | 1nmol |
| miRA1001610 | miDETECT A TrackTM hsa-miR-372-3p Forward Primer | 1nmol |
| miRA100262 | miDETECT A TrackTM hsa-miR-1323 Forward Primer | 1nmol |
| miRA100263 | miDETECT A TrackTM hsa-miR-520a-3p Forward Primer | 1nmol |
| miRA100281 | miDETECT A TrackTM hsa-miR-548ad-5p Forward Primer | 1nmol |
| miRA100266 | miDETECT A TrackTM hsa-miR-3614-5p Forward Primer | 1nmol |
| miRAN0002 | miDETECT A TrackTM U6 Forward Primer | 1nmol |
| miRAN0002-1-100 | miDETECT A Track U6 Forward Primer,100T | 1nmol |

Supplementary Table S6: Expression of the TOP 30 DE lncRNAs in neutrophil EXOs

| lncRNA / Simple RPKM | N1 | N2 | N3 | N4 | N5 | DM1 | DM2 | DM3 | DM4 | DM5 | log2(FC) | p-value |
| --- | --- | --- | --- | --- | --- | --- | --- | --- | --- | --- | --- | --- |
| ENST00000600489.1 | 0.00 | 0.61 | 0.00 | 0.00 | 0.00 | 5.97 | 8.13 | 1.39 | 7.89 | 0.00 | 5.432263 | 0.000071 |
| NR_136569.1 | 0.17 | 0.00 | 0.00 | 0.00 | 0.00 | 0.34 | 0.92 | 3.28 | 0.59 | 0.00 | 5.423278 | 0.000262 |
| ENST00000594492.1 | 0.00 | 1.88 | 0.52 | 0.79 | 1.69 | 10.03 | 0.00 | 6.46 | 14.27 | 13.44 | 3.713473 | 0.000019 |
| NR_003013.1 | 21.60 | 6.11 | 8.94 | 1.72 | 29.24 | 23.39 | 40.04 | 145.77 | 76.53 | 64.03 | 3.215967 | 0.000001 |
| ENST00000592523.1 | 1.56 | 1.91 | 2.62 | 0.00 | 0.43 | 2.75 | 7.27 | 6.30 | 10.37 | 8.21 | 3.20675 | 0.000027 |
| ENST00000607284.1 | 9.79 | 1.09 | 1.20 | 1.84 | 1.96 | 14.32 | 11.70 | 6.87 | 25.23 | 24.95 | 2.839373 | 0.000154 |
| NR_024393.1 | 0.22 | 0.54 | 0.89 | 2.96 | 0.24 | 2.21 | 4.10 | 2.16 | 11.30 | 1.54 | 2.632857 | 0.000970 |
| ENST00000526936.1 | 4.46 | 4.78 | 4.50 | 1.73 | 3.07 | 17.39 | 1.83 | 7.05 | 11.86 | 19.54 | 2.163156 | 0.000749 |
| ENST00000428280.1 | 2.91 | 5.80 | 6.37 | 8.28 | 0.00 | 12.09 | 11.57 | 11.50 | 16.13 | 5.10 | 1.970148 | 0.000835 |
| ENST00000419196.1 | 21.29 | 4.89 | 10.74 | 4.13 | 19.03 | 19.40 | 20.41 | 14.48 | 49.50 | 37.30 | 1.737177 | 0.000720 |
| ENST00000503469.2 | 5.77 | 8.83 | 22.30 | 7.45 | 12.69 | 24.65 | 23.69 | 14.17 | 24.26 | 22.73 | 1.577248 | 0.000732 |
| NR_135626.1 | 11.97 | 66.87 | 42.49 | 25.24 | 17.38 | 16.37 | 5.77 | 3.70 | 7.63 | 0.00 | -1.76398 | 0.000789 |
| ENST00000438753.1 | 28.09 | 26.13 | 18.21 | 18.89 | 21.59 | 12.25 | 3.71 | 2.85 | 0.00 | 2.37 | -1.89855 | 0.000106 |
| ENST00000450365.1 | 35.31 | 15.40 | 9.66 | 30.94 | 32.26 | 5.42 | 5.90 | 2.10 | 9.54 | 1.05 | -1.92895 | 0.000980 |
| NR_038194.1 | 16.18 | 38.66 | 20.67 | 36.80 | 51.62 | 13.83 | 1.77 | 3.98 | 11.47 | 0.00 | -2.01328 | 0.000948 |
| NR_123718.1 | 19.08 | 40.07 | 38.48 | 17.61 | 24.73 | 7.54 | 7.46 | 1.91 | 2.41 | 1.19 | -2.13623 | 0.000145 |
| NR_135530.1 | 15.64 | 13.07 | 38.25 | 18.37 | 30.50 | 9.29 | 3.12 | 1.50 | 2.52 | 0.00 | -2.33747 | 0.000417 |
| NR_040001.2 | 9.10 | 11.87 | 9.08 | 14.27 | 8.40 | 2.66 | 2.90 | 0.82 | 0.00 | 0.00 | -2.36788 | 0.000284 |
| ENST00000444796.1 | 23.76 | 44.79 | 31.95 | 24.56 | 26.13 | 16.54 | 0.00 | 1.92 | 3.24 | 0.00 | -2.43421 | 0.000643 |
| ENST00000453561.2 | 13.37 | 44.12 | 42.89 | 14.89 | 40.74 | 6.21 | 5.63 | 0.72 | 5.47 | 0.00 | -2.60922 | 0.000276 |
| ENST00000593824.1 | 12.10 | 31.67 | 4.44 | 27.29 | 19.96 | 3.32 | 1.81 | 0.77 | 0.97 | 0.00 | -3.24248 | 0.000019 |
| ENST00000503403.1 | 4.19 | 12.83 | 21.60 | 23.82 | 21.50 | 3.51 | 0.00 | 1.47 | 0.00 | 0.00 | -3.3745 | 0.000251 |
| ENST00000572850.1 | 21.72 | 33.88 | 20.36 | 8.85 | 14.48 | 0.00 | 1.44 | 1.85 | 0.00 | 0.00 | -3.7555 | 0.000019 |
| NR_110815.1 | 2.52 | 1.66 | 2.61 | 2.20 | 2.98 | 0.19 | 0.00 | 0.00 | 0.34 | 0.00 | -4.13723 | 0.000534 |
| ENST00000519840.1 | 16.53 | 16.02 | 10.86 | 3.58 | 10.57 | 1.16 | 0.42 | 0.27 | 0.00 | 0.00 | -4.25406 | 0.000004 |
| ENST00000587907.1 | 8.38 | 1.50 | 4.40 | 9.51 | 2.47 | 0.21 | 0.22 | 0.14 | 0.36 | 0.00 | -4.27549 | 0.000022 |
| NR_104232.1 | 1.64 | 4.34 | 6.23 | 3.10 | 2.40 | 0.00 | 0.00 | 0.19 | 0.00 | 0.00 | -4.64883 | 0.000205 |
| ENST00000581910.1 | 0.00 | 16.36 | 8.51 | 12.35 | 7.73 | 0.71 | 0.00 | 0.00 | 0.00 | 0.00 | -4.66708 | 0.000802 |
| ENST00000592296.1 | 8.63 | 9.82 | 20.73 | 19.12 | 2.71 | 0.00 | 0.00 | 0.43 | 0.00 | 0.00 | -5.27352 | 0.000012 |
| ENST00000577199.1 | 6.80 | 16.66 | 9.68 | 6.20 | 4.40 | 0.00 | 0.00 | 0.00 | 0.00 | 0.00 | -5.98241 | 0.000000 |

Supplementary Table S7: TOP 29 GO terms of DE lncRNAs target genes

| ID | Description | GeneRatio | pvalue | Cluster |
| --- | --- | --- | --- | --- |
| GO:0032648 | regulation of interferon-beta production | 8/1172 | 0.011244 | Up |
| GO:0032608 | interferon-beta production | 8/1172 | 0.014208 | Up |
| GO:0072641 | type I interferon secretion | 3/1172 | 0.021764 | Up |
| GO:0032479 | regulation of type I interferon production | 13/1172 | 0.026579 | Up |
| GO:0032606 | type I interferon production | 13/1172 | 0.028328 | Up |
| GO:0032728 | positive regulation of interferon-beta production | 5/1172 | 0.043594 | Up |
| GO:0032688 | negative regulation of interferon-beta production | 3/1172 | 0.054673 | Up |
| GO:0001913 | T cell mediated cytotoxicity | 8/1172 | 0.012663 | Up |
| GO:0032755 | positive regulation of interleukin-6 production | 11/1172 | 0.012967 | Up |
| GO:0070102 | interleukin-6-mediated signaling pathway | 4/1172 | 0.019655 | Up |
| GO:0032675 | regulation of interleukin-6 production | 14/1172 | 0.023819 | Up |
| GO:0032635 | interleukin-6 production | 14/1172 | 0.040253 | Up |
| GO:0014842 | regulation of skeletal muscle satellite cell proliferation | 3/1172 | 0.045018 | Up |
| GO:0014841 | skeletal muscle satellite cell proliferation | 3/1172 | 0.054673 | Up |
| GO:0032648 | regulation of interferon-beta production | 11/1560 | 0.002158 | Down |
| GO:0032608 | interferon-beta production | 11/1560 | 0.00302 | Down |
| GO:0035456 | response to interferon-beta | 7/1560 | 0.007313 | Down |
| GO:0032688 | negative regulation of interferon-beta production | 4/1560 | 0.025411 | Down |
| GO:0035457 | cellular response to interferon-alpha | 4/1560 | 0.006998 | Down |
| GO:0035455 | response to interferon-alpha | 5/1560 | 0.022533 | Down |
| GO:0032480 | negative regulation of type I interferon production | 8/1560 | 0.032761 | Down |
| GO:0014857 | regulation of skeletal muscle cell proliferation | 4/1560 | 0.032392 | Down |
| GO:0014856 | skeletal muscle cell proliferation | 4/1560 | 0.040384 | Down |
| GO:0055001 | muscle cell development | 24/1560 | 0.013034 | Down |
| GO:0014842 | regulation of skeletal muscle satellite cell proliferation | 4/1560 | 0.019421 | Down |
| GO:0014841 | skeletal muscle satellite cell proliferation | 4/1560 | 0.025411 | Down |
| GO:0032675 | regulation of interleukin-6 production | 18/1560 | 0.014954 | Down |
| GO:0032755 | positive regulation of interleukin-6 production | 12/1560 | 0.038491 | Down |
| GO:0032635 | interleukin-6 production | 18/1560 | 0.028706 | Down |

Supplementary Table S8: TOP 24 KEGG pathways of DE lncRNAs target genes

| ID | Description | GeneRatio | pvalue | Cluster |
| --- | --- | --- | --- | --- |
| hsa04659 | Th17 cell differentiation | 10/547 | 0.206363 | Up |
| hsa04620 | Toll-like receptor signaling pathway | 5/547 | 0.856458 | Up |
| hsa04390 | Hippo signaling pathway | 16/547 | 0.067096 | Up |
| hsa04630 | JAK-STAT signaling pathway | 15/547 | 0.153127 | Up |
| hsa04150 | mTOR signaling pathway | 13/547 | 0.262123 | Up |
| hsa04550 | Signaling pathways regulating pluripotency of stem cells | 18/547 | 0.007834 | Up |
| hsa04210 | Apoptosis | 13/547 | 0.147849 | Up |
| hsa04144 | Endocytosis | 24/547 | 0.049163 | Up |
| hsa04152 | AMPK signaling pathway | 7/547 | 0.735279 | Up |
| hsa04068 | FoxO signaling pathway | 15/547 | 0.037017 | Up |
| hsa04010 | MAPK signaling pathway | 17/547 | 0.820928 | Up |
| hsa04151 | PI3K-Akt signaling pathway | 29/547 | 0.195877 | Up |
| hsa04659 | Th17 cell differentiation | 13/686 | 0.135837 | Down |
| hsa04620 | Toll-like receptor signaling pathway | 13/686 | 0.115763 | Down |
| hsa04630 | JAK-STAT signaling pathway | 23/686 | 0.012648 | Down |
| hsa04150 | mTOR signaling pathway | 14/686 | 0.461705 | Down |
| hsa04550 | Signaling pathways regulating pluripotency of stem cells | 18/686 | 0.059177 | Down |
| hsa04210 | Apoptosis | 18/686 | 0.046776 | Down |
| hsa04144 | Endocytosis | 25/686 | 0.21401 | Down |
| hsa04217 | Necroptosis | 22/686 | 0.023289 | Down |
| hsa04152 | AMPK signaling pathway | 9/686 | 0.726958 | Down |
| hsa04068 | FoxO signaling pathway | 14/686 | 0.246989 | Down |
| hsa04010 | MAPK signaling pathway | 32/686 | 0.11093 | Down |
| hsa04151 | PI3K-Akt signaling pathway | 38/686 | 0.099564 | Down |

Supplementary Table S9: Expression of the TOP 32 DE miRNAs in neutrophil EXOs

| MiRNA / Simple RPM | N1 | N2 | N3 | N4 | N5 | DM1 | DM2 | DM3 | DM4 | DM5 | log2(FC) | P-value |
| --- | --- | --- | --- | --- | --- | --- | --- | --- | --- | --- | --- | --- |
| hsa-miR-3614-5p | 0 | 26.85 | 0 | 36.7 | 79.48 | 415.24 | 129.94 | 1480.97 | 350.65 | 367.57 | 4.2621 | 8.27E-06 |
| hsa-miR-4792 | 60.26 | 322.19 | 363.27 | 440.4 | 688.85 | 166.09 | 0 | 69.42 | 0 | 0 | -2.993 | 0.002083 |
| hsa-miR-1323 | 60.26 | 80.55 | 290.61 | 1578.1 | 291.44 | 138.41 | 43.31 | 0 | 36.91 | 81.68 | -2.9377 | 0.004838 |
| hsa-miR-516a-5p | 120.53 | 26.85 | 145.31 | 1761.6 | 132.47 | 110.73 | 14.44 | 23.14 | 55.37 | 0 | -3.4244 | 0.005303 |
| hsa-miR-1180-3p | 0 | 0 | 0 | 0 | 26.49 | 193.78 | 245.44 | 46.28 | 18.46 | 0 | 4.2498 | 0.006997 |
| hsa-miR-512-3p | 30.13 | 107.4 | 145.31 | 1357.9 | 52.99 | 55.36 | 14.44 | 23.14 | 55.37 | 40.84 | -3.1626 | 0.008339 |
| hsa-miR-372-3p | 60.26 | 0 | 0 | 146.8 | 79.48 | 0 | 0 | 0 | 0 | 0 | -12.4845 | 0.010458 |
| hsa-miR-451a | 11510.53 | 6712.31 | 2688.17 | 6605.99 | 19764.73 | 4899.79 | 64882.19 | 8932.08 | 42557.9 | 32918.11 | 1.7054 | 0.014249 |
| hsa-miR-23a-5p | 0 | 53.7 | 0 | 0 | 26.49 | 83.05 | 28.88 | 370.24 | 36.91 | 81.68 | 2.9053 | 0.0143 |
| hsa-miR-183-5p | 512.25 | 134.25 | 72.65 | 146.8 | 317.93 | 442.92 | 606.38 | 439.66 | 682.85 | 735.14 | 1.296 | 0.015442 |
| hsa-miR-486-3p | 0 | 80.55 | 0 | 36.7 | 105.98 | 55.36 | 259.88 | 23.14 | 313.74 | 449.25 | 2.3027 | 0.015709 |
| hsa-miR-223-5p | 1024.5 | 1020.27 | 5739.61 | 4770.99 | 4583.51 | 6699.15 | 4663.32 | 15665.86 | 9116.91 | 5636.1 | 1.2856 | 0.019612 |
| hsa-miR-4488 | 0 | 966.57 | 0 | 36.7 | 264.94 | 83.05 | 0 | 0 | 0 | 0 | -3.9327 | 0.021914 |
| hsa-miR-1268a | 0 | 0 | 0 | 0 | 0 | 55.36 | 57.76 | 92.56 | 0 | 0 | 12.0062 | 0.022168 |
| hsa-let-7f-1-3p | 30.13 | 53.7 | 72.65 | 36.7 | 26.49 | 0 | 0 | 0 | 0 | 0 | -12.1011 | 0.026657 |
| hsa-miR-520a-3p | 150.66 | 0 | 72.65 | 1284.5 | 105.98 | 110.73 | 28.88 | 23.14 | 36.91 | 81.68 | -2.5201 | 0.029658 |
| hsa-miR-424-3p | 241.06 | 107.4 | 72.65 | 183.5 | 158.97 | 636.7 | 577.5 | 138.84 | 147.64 | 367.57 | 1.2908 | 0.030296 |
| hsa-miR-16-2-3p | 271.19 | 349.04 | 435.92 | 550.5 | 397.41 | 193.78 | 1140.56 | 555.36 | 1144.23 | 1061.87 | 1.0312 | 0.031251 |
| hsa-miR-424-5p | 331.46 | 214.79 | 72.65 | 110.1 | 105.98 | 27.68 | 28.88 | 46.28 | 110.73 | 0 | -1.967 | 0.032729 |
| hsa-miR-363-3p | 602.65 | 107.4 | 145.31 | 256.9 | 370.92 | 304.51 | 1429.31 | 277.68 | 498.29 | 1388.61 | 1.3942 | 0.033039 |
| hsa-miR-122-5p | 4610.24 | 19975.84 | 50421.39 | 53214.91 | 59453.16 | 58991.25 | 41392.35 | 53430.52 | 158992.3 | 133877.9 | 1.251 | 0.035943 |
| hsa-miR-1278 | 0 | 0 | 0 | 0 | 0 | 0 | 28.88 | 46.28 | 0 | 122.52 | 11.949 | 0.038539 |
| hsa-miR-542-3p | 361.59 | 241.64 | 0 | 220.2 | 79.48 | 0 | 28.88 | 46.28 | 92.28 | 40.84 | -2.1161 | 0.039057 |
| hsa-miR-4433b-5p | 0 | 134.25 | 72.65 | 0 | 26.49 | 0 | 0 | 0 | 0 | 0 | -12.1885 | 0.039328 |
| hsa-miR-1307-5p | 30.13 | 187.94 | 72.65 | 36.7 | 0 | 0 | 28.88 | 0 | 0 | 0 | -3.503 | 0.043337 |
| hsa-miR-548ad-5p | 0 | 0 | 0 | 0 | 26.49 | 0 | 86.64 | 92.56 | 0 | 81.68 | 3.2999 | 0.043519 |
| hsa-miR-548ae-5p | 0 | 0 | 0 | 0 | 26.49 | 0 | 86.64 | 92.56 | 0 | 81.68 | 3.2999 | 0.043523 |
| hsa-miR-182-5p | 1114.89 | 375.89 | 217.96 | 587.2 | 582.87 | 525.97 | 1588.13 | 1157.01 | 609.02 | 2287.11 | 1.0992 | 0.045323 |
| hsa-miR-195-5p | 512.25 | 349.04 | 145.31 | 403.7 | 238.45 | 138.41 | 144.38 | 92.56 | 203.01 | 0 | -1.5113 | 0.046112 |
| hsa-miR-27b-3p | 40347.12 | 15921.6 | 1816.33 | 14863.48 | 10677.19 | 7086.7 | 8532.57 | 2429.71 | 7622.04 | 1306.92 | -1.6322 | 0.046586 |
| hsa-miR-518e-3p | 30.13 | 26.85 | 0 | 550.5 | 0 | 27.68 | 0 | 0 | 18.46 | 0 | -3.7187 | 0.046622 |
| hsa-miR-1273h-3p | 0 | 26.85 | 0 | 0 | 0 | 0 | 57.75 | 23.14 | 73.82 | 81.68 | 3.1382 | 0.049429 |

Supplementary Table S10: TOP 28 GO terms of DE miRNAs target genes

| ID | Description | GeneRatio | pvalue | Cluster |
| --- | --- | --- | --- | --- |
| GO:0051017 | actin filament bundle assembly | 27/1939 | 0.003946 | Down |
| GO:0007015 | actin filament organization | 63/1939 | 0.000539 | Down |
| GO:0110053 | regulation of actin filament organization | 46/1939 | 0.000272 | Down |
| GO:0032956 | regulation of actin cytoskeleton organization | 62/1939 | 0.000004 | Down |
| GO:0032970 | regulation of actin filament-based process | 69/1939 | 0.000003 | Down |
| GO:1901861 | regulation of muscle tissue development | 34/1939 | 0.000013 | Down |
| GO:0048634 | regulation of muscle organ development | 34/1939 | 0.000017 | Down |
| GO:0060537 | muscle tissue development | 71/1939 | 0.000004 | Down |
| GO:0048641 | regulation of skeletal muscle tissue development | 13/1939 | 0.002245 | Down |
| GO:0061028 | establishment of endothelial barrier | 13/1939 | 0.000141 | Down |
| GO:0003158 | endothelium development | 30/1939 | 0.000011 | Down |
| GO:0045446 | endothelial cell differentiation | 27/1939 | 0.000023 | Down |
| GO:0001885 | endothelial cell development | 17/1939 | 0.000062 | Down |
| GO:0043405 | activation of MAPK activity | 54/1939 | 0.000897 | Down |
| GO:0051017 | actin filament bundle assembly | 46/2578 | 0.000000 | Up |
| GO:0061572 | actin filament bundle organization | 47/2578 | 0.000000 | Up |
| GO:0007015 | actin filament organization | 99/2578 | 0.000000 | Up |
| GO:0001885 | endothelial cell development | 17/2578 | 0.001812 | Up |
| GO:0045446 | endothelial cell differentiation | 30/2578 | 0.000207 | Up |
| GO:0003158 | endothelium development | 33/2578 | 0.000167 | Up |
| GO:0007517 | muscle organ development | 86/2578 | 0.000028 | Up |
| GO:0060537 | muscle tissue development | 96/2578 | 0.000000 | Up |
| GO:0042692 | muscle cell differentiation | 81/2578 | 0.000025 | Up |
| GO:0045445 | myoblast differentiation | 22/2578 | 0.002512 | Up |
| GO:0045766 | positive regulation of angiogenesis | 45/2578 | 0.000667 | Up |
| GO:0045765 | regulation of angiogenesis | 77/2578 | 0.000428 | Up |
| GO:0030178 | negative regulation of Wnt signaling pathway | 39/2578 | 0.000476 | Up |
| GO:0000187 | activation of MAPK activity | 38/2578 | 0.000143 | Up |

Supplementary Table S11: TOP 33 KEGG pathways of DE miRNAs target genes

| ID | Description | GeneRatio | pvalue | Cluster |
| --- | --- | --- | --- | --- |
| hsa04062 | Chemokine signaling pathway | 34/1174 | 0.133370 | Up |
| hsa04144 | Endocytosis | 53/1174 | 0.002100 | Up |
| hsa04810 | Regulation of actin cytoskeleton | 49/1174 | 0.000926 | Up |
| hsa04660 | T cell receptor signaling pathway | 21/1174 | 0.078432 | Up |
| hsa04350 | TGF-beta signaling pathway | 29/1174 | 0.000060 | Up |
| hsa04659 | Th17 cell differentiation | 25/1174 | 0.012648 | Up |
| hsa04668 | TNF signaling pathway | 26/1174 | 0.012114 | Up |
| hsa04370 | VEGF signaling pathway | 14/1174 | 0.047501 | Up |
| hsa04310 | Wnt signaling pathway | 38/1174 | 0.001826 | Up |
| hsa04150 | mTOR signaling pathway | 34/1174 | 0.009188 | Up |
| hsa00010 | Glycolysis / Gluconeogenesis | 14/1174 | 0.125159 | Up |
| hsa04330 | Notch signaling pathway | 17/1174 | 0.000573 | Up |
| hsa04068 | FoxO signaling pathway | 40/1174 | 0.000003 | Up |
| hsa04010 | MAPK signaling pathway | 66/1174 | 0.000303 | Up |
| hsa04151 | PI3K-Akt signaling pathway | 81/1174 | 0.000028 | Up |
| hsa04152 | AMPK signaling pathway | 36/1174 | 0.000016 | Up |
| hsa04062 | Chemokine signaling pathway | 34/900 | 0.004436 | Down |
| hsa04144 | Endocytosis | 39/900 | 0.016530 | Down |
| hsa04810 | Regulation of actin cytoskeleton | 45/900 | 0.000025 | Down |
| hsa04660 | T cell receptor signaling pathway | 25/900 | 0.000171 | Down |
| hsa04350 | TGF-beta signaling pathway | 22/900 | 0.000704 | Down |
| hsa04659 | Th17 cell differentiation | 21/900 | 0.008607 | Down |
| hsa04668 | TNF signaling pathway | 25/900 | 0.000669 | Down |
| hsa04370 | VEGF signaling pathway | 12/900 | 0.031943 | Down |
| hsa04310 | Wnt signaling pathway | 31/900 | 0.002008 | Down |
| hsa04150 | mTOR signaling pathway | 30/900 | 0.001943 | Down |
| hsa04630 | JAK-STAT signaling pathway | 25/900 | 0.070351 | Down |
| hsa04620 | Toll-like receptor signaling pathway | 17/900 | 0.079522 | Down |
| hsa04210 | Apoptosis | 21/900 | 0.090838 | Down |
| hsa04152 | AMPK signaling pathway | 34/900 | 0.000000 | Down |
| hsa04068 | FoxO signaling pathway | 34/900 | 0.000003 | Down |
| hsa04010 | MAPK signaling pathway | 76/900 | 0.000000 | Down |
| hsa04151 | PI3K-Akt signaling pathway | 66/900 | 0.000029 | Down |
