# Supplementary figures and images for: The Functional Roles of RNAs Cargoes Released by Neutrophil-Derived Exosomes in Dermatomyositis
Source: Front Pharmacol. 2021 Sep 17;12:727901. doi: 10.3389/fphar.2021.727901 (PMC8484304; doi:10.3389/fphar.2021.727901)

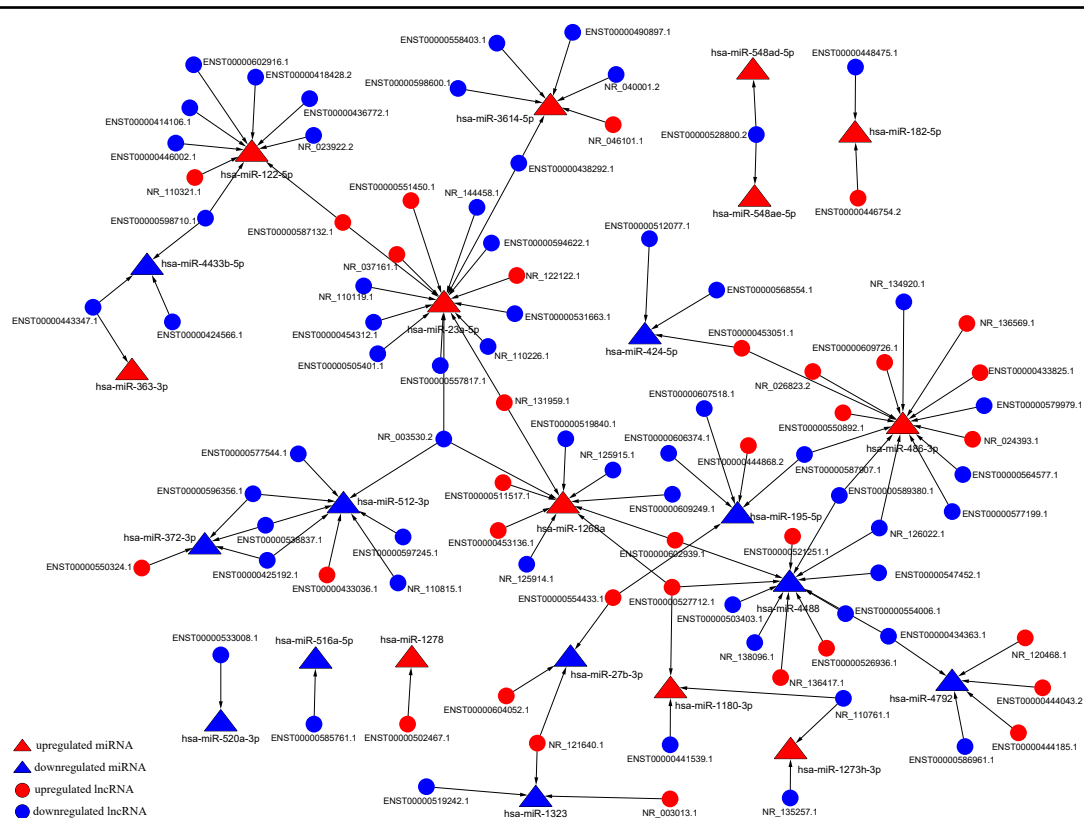

Supplement: Supplementary file 2 [file Image2.PDF]

# Supplementary figure 3

A **log2(FC) value of DE lncRNAs**

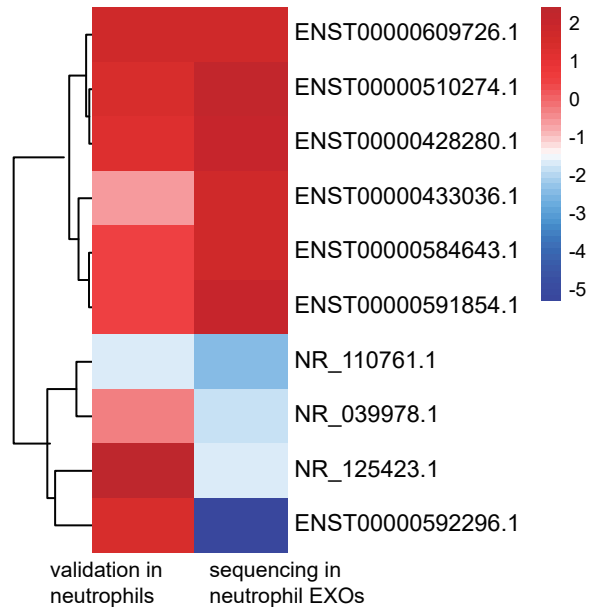

B **log2(FC) value of DE miRNAs**

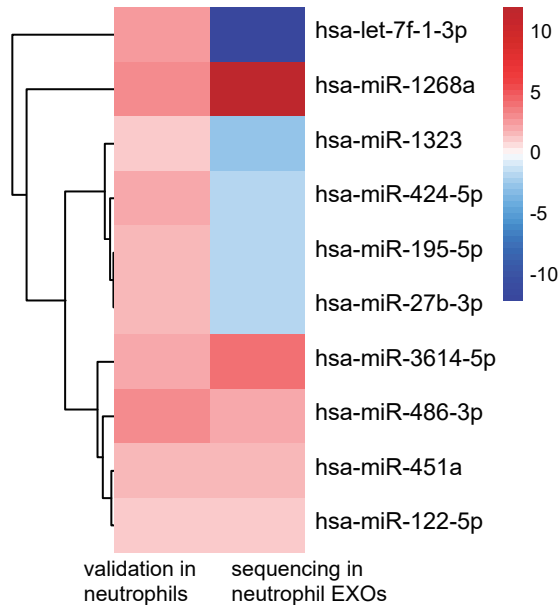

Supplement: Supplementary file 3 [file Image3.PDF]
