# Supplementary material for: The Functional Roles of RNAs Cargoes Released by Neutrophil-Derived Exosomes in Dermatomyositis
Source: Front Pharmacol. 2021 Sep 17;12:727901. doi: 10.3389/fphar.2021.727901 (PMC8484304; doi:10.3389/fphar.2021.727901)

Supplementary figure 1A

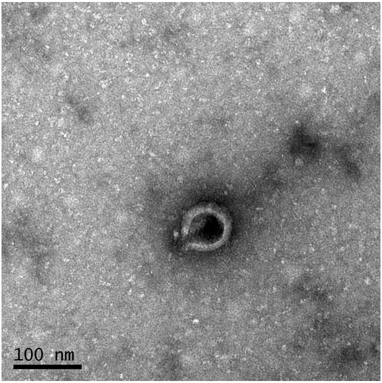

B

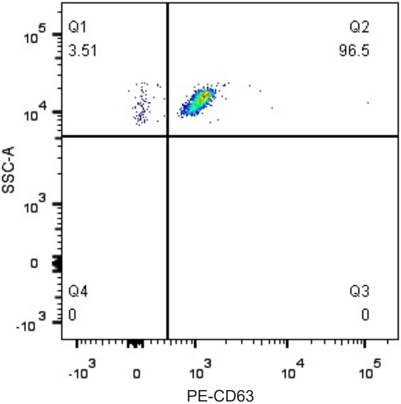

C

Temperature (°C):	25.0	Duration Used (s):	50
Count Rate (kcps):	100.3	Measurement Position (mm):	4.65
Cell Description:	Disposable sizing cuvette	Attenuator:	11

			Size (d.nm):	% Intensity:	St Dev (d.nm):
Z-Average (d.nm):	132.7	Peak 1:	294.8	83.4	134.3
PdI:	0.614	Peak 2:	16.43	8.9	5.605
Intercept:	0.974	Peak 3:	49.64	7.7	15.36

Result quality : Refer to quality report

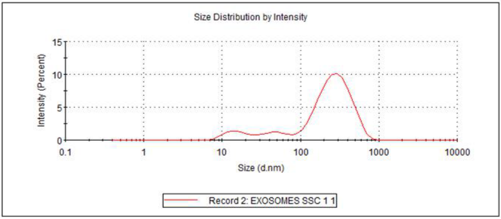

Supplement: Supplementary file 5 [file Image1.PDF]
